# Review of "Biomedical Nanotechnology", edited by Neelina H. Malsch

**DOI:** 10.1186/1475-925X-5-20

**Published:** 2006-03-22

**Authors:** Eduardo Abreu

**Affiliations:** 1 Childrens' Hospital of Boston, Department of Orthopaedic Surgery, Enders 1022, 300 Longwood Street, Boston MA 02115, USA

With the recent rise in interest on potential nanotechnology applications to biomedical research, books dedicated to this novel field are not only expected but welcome. *Biomedical Nanotechnology *(edited by Neelina H. Malsch) is one of these books which, hopefully, will continue to encourage the growth of this promising discipline. The book is organized in such a way that it blends technical and scientific information (especially as related to three subfields: nanodrugs and drug delivery; prostheses and implants; and diagnostics and screening technologies for laboratory use) with social and economic analyzes of the field's recent and anticipated future impact to the world's health needs.

The book is divided into 7 chapters, written by 15 expert contributors. In the introduction, it looks into the possible interactions of nanotechnology and biomedicine, and discusses how these two converging technologies can be amalgamated to create a new interdisciplinary field. As part of this discussion, concepts such as nanotechnology and nanobiomedicine are precisely defined. In the introduction there is also a brief but useful description of the following chapters.

Chapter 1 summarizes the current *status quo *of the field worldwide (more specifically, USA, Europe and Japan). Although this chapter is quite comprehensive and detailed, there is the risk that in such fast developing field this kind of data will become rapidly outdated. Furthermore, this chapter could be enriched by the inclusion of a short summary about other countries. For example, are there any signs of biomedical nanothechnology initiatives in South America or in Asian countries other than Japan? Despite that, the analyzes in chapter 1 can be successfully used as models for those interested in producing similar work, either more updated or focusing in other countries or regions of the world. Chapters 2 through 4 describe the state of the art of biomedical nanotechnology research in 3 important subfields, already mentioned: drug delivery; prostheses and implants; and diagnostics and screening technologies. These are excellent chapters that have as merit the explanation of quite complex concepts in a simple manner. The figures and pictures are extraordinarily simple (but of good quality) and helpful, and, along with excellent tables, complement the straightforward style of these chapters. The following chapters discuss the role of nanotechnology for biodefense, a very opportune topic, and the social and economic aspects related to the emergence of biomedical nanotechnology. The final chapter focuses on safety and regulatory issues. The references at the end of each chapter are very useful for those interested in expand their knowledge in a particular topic. Also helpful is the general index at the end of the book.

The targeted audience is not only nanoscience researchers, but also those involved in non governmental organizations, students and even lay person who might have an interest in the field. In summary, this is a very well written book, accessible to those with and without expertise in the area of nanotechnology, that will certainly prove to be a valuable tool for those interested in the development of this new, interdisciplinary and exciting field.

